# Assisted living resident quality of life questionnaire: development and validation

**DOI:** 10.1186/s41687-026-00994-6

**Published:** 2026-01-24

**Authors:** Catherine H. Coddington, Anna M. Kimura, Marissa Hughes, Tetyana P. Shippee, Timothy J. Beebe, Rachel Shands

**Affiliations:** 1 Vital Research, 6300 Wilshire Boulevard, Suite 860, Los Angeles, CA 90048 USA; 2 Division of Health Policy and Management, School of Public Health, University of Minnesota, 420 Delaware St SE, MMC 729 Mayo, Minneapolis, MN 55455 USA; 3 Minnesota Department of Human Services, PO Box 64976, St. Paul, MN 55164-0976 USA

**Keywords:** Instrument development, Measurement validation, Long-term care, Assisted living, Quality of life, Person centered, Stakeholder engagement, Patient centeredness

## Abstract

**Background:**

As an increasing number of older adults in the U.S. seek out assisted living services, there is a continued need to comprehensively assess quality within assisted living facilities to ensure consumers are well-informed when deciding where to seek care, and to assist quality improvement efforts within facilities. The current study aimed to develop, test, and validate a questionnaire to holistically measure quality of life among assisted living residents, which is one metric for quality in assisted living.

**Methodology:**

The questionnaire development, testing, and validation process included three phases. First, informed by existing literature, an item bank was created and then refined based on stakeholder feedback and cognitive interviews with assisted living residents. Second, targeted pilot testing was completed via mailed questionnaires and in-person cognitive interviews with assisted living residents in memory care units. Third, pilot testing across Minnesota, via in-person, phone, and mail administration, was conducted to test the reliability and validity of the measure.

**Results:**

Factor analysis results revealed five subscales: The People Who Work Here; Food; Security, Safety & Privacy; Choice/Autonomy; Religion/Spirituality. Two additional sub-domains were identified: Activities and Finances. All sub-scales indicated adequate to high internal consistency and were positively correlated with other indicators of resident satisfaction in expected ways. Measurement equivalence across administration modes (mail, phone, in-person) suggests inter-changeability.

**Conclusions:**

The questionnaire developed and tested in this study to measure resident quality of life in assisted living facilities is a valid and reliable tool that can be used in large-scale measurement efforts to capture one aspect of quality at the facility level.

**Supplementary Information:**

The online version contains supplementary material available at 10.1186/s41687-026-00994-6.

## Background

In 2020, about 55.8 million people or 16.8% of the U.S. population were aged 65 and older, with the older adult population projected to grow through 2030 [1]. As the older adult population grows, long-term care options like assisted living facilities (ALFs) are increasingly important in the care continuum for older adults. ALFs provide housing and services to support older adults’ daily care needs such as personal care, 24-hour supervision, meals, and managing medications, while encouraging residents’ independence [10]. Some ALFs provide specialized services such as dementia or memory care [11]. In the U.S., there are about 30,600 ALFs with 1,197,600 licensed units or beds, serving about 818,800 people [11].

ALFs have become increasingly popular due to the lower costs compared to nursing homes and their emphasis on person-centered care [19]. However, the quality of ALFs can vary widely as there are no federal regulations, and only a few states mandate standards of care [11]. Workforce challenges such as staff shortages and low wages can also affect the quality of care [19]. We define quality as “the capacity to satisfy the needs and wants of the users of a service or product” [5]. It is necessary to assess the quality of ALFs to improve residents’ safety and physical and mental health, and ensure older adults have access to information on care options [18, 19].

Despite growing research on quality measurement in long-term care [3], there is a need to develop holistic measures that incorporate several aspects of quality. Shippee and colleagues [14], conducted a scoping review and identified nine key domains of quality for ALFs, which included: (1) resident quality of life (QOL); (2) resident/family satisfaction; (3) staffing and staff-related outcomes; (4) resident safety; (5) resident health outcomes; (6) care planning and integration; (7) physical and social environment; (8) service availability; and (9) core values and philosophy. They also identified a lack of standardized, resident-reported quality measures in assisted living. To address this gap, this paper aims to develop a holistic questionnaire to assess resident-reported QOL in ALFs and to assess reliability and validity of the measure via pilot testing.

## Methods

### Questionnaire development

**Fig. 1:**
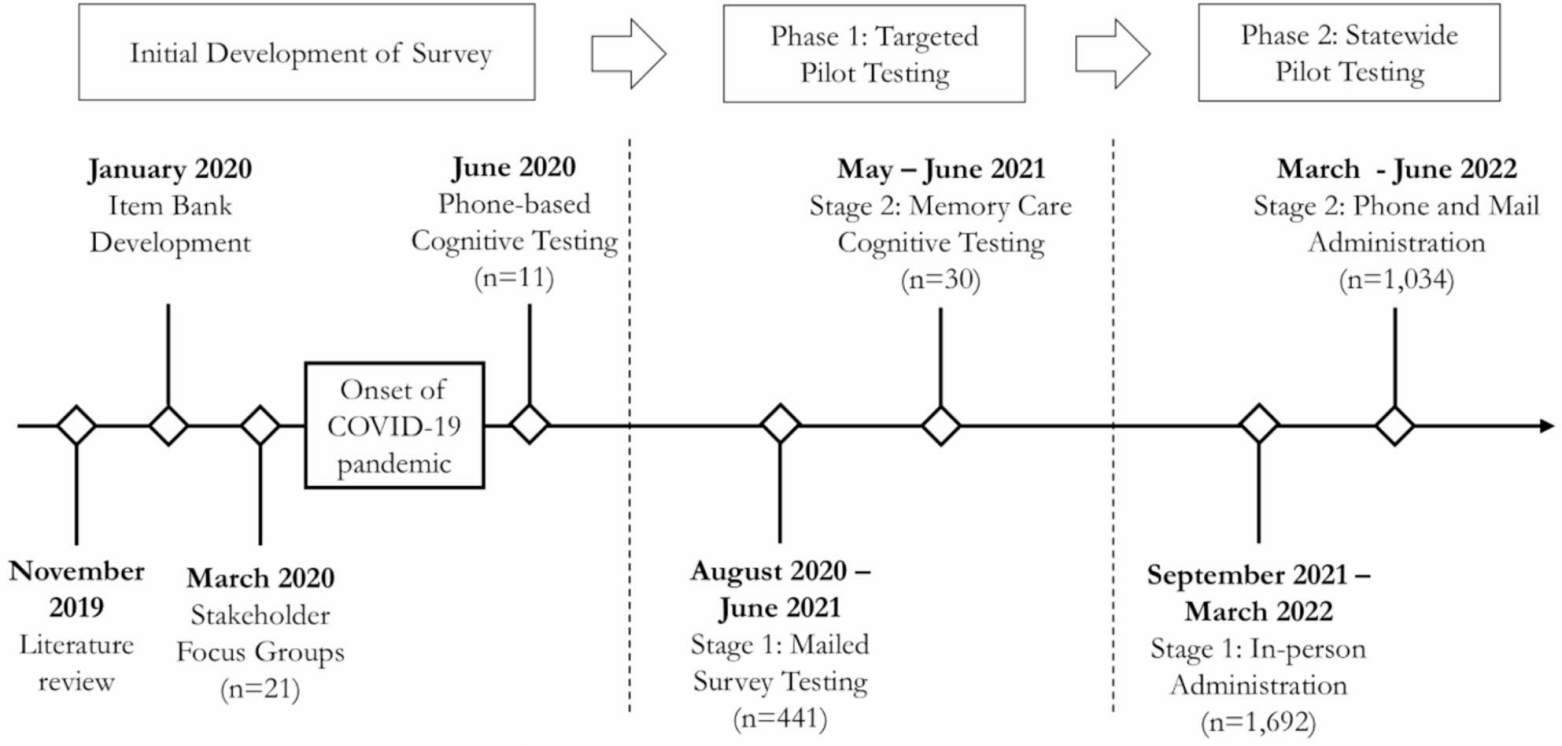
Timeline for Survey Development and Pilot Testing

In this section, we outline the initial process of developing the resident QOL questionnaire, then describe Phase 1 of targeted pilot testing, followed by Phase 2 of statewide pilot testing (see Fig. 1 for timeline). Questionnaire development spanned two years.

### Initial development

The initial development of the resident QOL questionnaire was informed by a study conducted by Shippee and colleagues [13] which identified the following domains as particularly important for the Minnesota context: resident QOL, family satisfaction, staff quality, and safety. Minnesota’s Department of Human Services (DHS) commissioned this study to develop, pilot, and psychometrically test an assisted living resident QOL questionnaire with the goal of developing assisted living report cards to inform consumers on the quality of different settings. While the questionnaire was developed and tested in Minnesota, given the need for more holistic measures of quality within assisted living communities, we aimed to develop a questionnaire that could be used across the U.S.

### Item bank

We created an item bank starting with the domains and instruments identified by Shippee et al. [13] and entered 439 items into a spreadsheet. For each item we included the response scale, respondent, typical administration mode, and domain as listed by the instrument developers. A team of 3–4 researchers assessed each item and removed redundant and low-quality items. For example, several items asked about getting around the facility with ease, but in slightly different ways.

The remaining items were linked to the domains identified as important for Minnesota’s context (see Shippee et al. [13]) to ensure there were several high-quality items for each domain. Some items were rewritten for clarity (e.g., shortened, removing hard-to-hear words like ‘staff’, etc.) given the target population. Items were adjusted for a frequency-based response scale and to account for residents being the respondent rather than a family or staff member. The initial questionnaire was shared with the project team including Minnesota DHS staff and researchers at the University of Minnesota. This team engaged in three rounds of revisions until a draft questionnaire was ready to share with additional stakeholders. Of the items included in the item bank, 42 were in the version of the survey shared with stakeholders for feedback.

### Stakeholder focus groups

Two focus groups were conducted in March 2020. One focus group was held with 11 assisted living providers operating in Minnesota and the other with 10 representatives of advocacy groups. Providers worked in small, medium, and large facilities, and multi-facility chains. Representatives of advocacy groups were from a range of organizations from family facing to those focused on serving people with mental health needs. Feedback focused on questionnaire length and revisions for specific items mostly related to word choice and terminology. Participants gave general feedback about the diversity of residents living in ALFs throughout the state, ranging from those who are completely independent to those receiving more extensive services. This feedback suggested that some items were relevant for some but likely not all residents (e.g., an item related to medication management).

### Phone-based cognitive testing

The next version of the questionnaire was tested through cognitive interviews with residents in June 2020 via phone-based administration (in-person interviews were not possible due to the COVID-19 pandemic). Cognitive interviews are designed to evaluate and correct sources of response error and provide a unique understanding of the cognitive processes involved in responding to the draft instrument [9, 17]. Interview results (*n* = 11) led to questionnaire modifications, including selecting the 3- versus 4-point response scale since several participants experienced challenges or could not respond using the 4-point scale. Further, when the instrument was tested with residents receiving memory care services (described below) responses were more varied when the 3- versus 4-point scale was used. Cognitive testing also helped to identify redundant items and items needing additional probes such as an item asking if the people who work in the facility do things the way the respondent liked.

### Phase 1: Targeted pilot testing

Phase 1 of targeted pilot testing included two stages (Fig. 1). Stage one was conducted between August 2020 to January 2021 via mailed questionnaires. Stage two included cognitive testing with residents receiving memory care services in May 2021. Testing the questionnaire with residents receiving memory care services was critical because approximately one third of all ALF residents in Minnesota had a diagnosis of dementia at the time of phase one pilot testing.

### Stage 1: Mailed questionnaire testing

#### Procedures

For initial pilot testing, we invited a subset of 150 facilities that were randomly sampled across the following strata: facilities of differing capacities (i.e., 6–15 residents; 16–50 residents; and more than 50 residents) and those located in urban and non-urban areas. Participation rates for the sampled facilities were low, so participation was opened to all facilities with more than five residents in September 2020. Ten different counties across the state were included at this stage of testing and no facilities were on tribal lands. Initially, participating facilities provided census lists of all residents to the research team, and we sent individualized questionnaire packets to each resident. A few months into data collection, to ensure resident privacy, we sent a questionnaire package to each facility and asked the staff to distribute packets to each resident. Questionnaires were distributed to 1,649 individuals. Residents could complete the questionnaire by mail or phone.

#### Participant characteristics

Completed questionnaires were received from 441 residents (27% response rate) in 46 different facilities. About half of the participating facilities were in rural areas. Fourteen facilities had occupancies of 6–15 residents, 21 had 16–50 residents, and seven had more than 50 residents. Residents (*M*_*age*_=85, range = 40–104 years) who participated in the mailed questionnaire pilot testing were mostly female (72%) and almost all identified as White/Caucasian (94%).

#### Piloted questionnaire

The tested questionnaire included 57 items grouped into the following nine domains plus a demographics section: The People Who Work Here (10-items); Physical Environment (3-items); Food (6-items); Meaning Activities/Social Engagement (6-items); Choice/Autonomy (5-items); Religion/Spirituality (3-items); Security, Safety, and Privacy (6-items); Finances (3-items); and Overall Rating (2-items). Respondents selected one of the following options to answer each item: ‘Yes, always/most of the time,’ ‘Yes, some of the time,’ ‘No, rarely or never.’ and ‘Not applicable/Don’t know.’

### Stage 2: Memory care cognitive testing

#### Procedures

Because this component of testing was done in-person, sites were purposefully selected based on their proximity to Minneapolis and St. Paul. All residents in memory care units were eligible to participate, unless they were in isolation or quarantine or if a legal guardian declined on their behalf. Two interviewers, each with several years of experience conducting in-person interviews with residents of nursing homes and ALFs, visited one facility per day. Four ALFs with memory care units or buildings agreed to participate.

#### Characteristics of study participants

Thirty residents completed cognitive testing interviews out of the 52 eligible residents who were approached. Demographics were missing for 12–18% of respondents. However, 58% identified as female and 27% as male. 49% of respondents identified as White/Caucasian, 21% self-described with an identity not listed in the response categories, and 6% preferred not to answer.

#### Piloted questionnaire

Two different questionnaire forms were used to test the 3- and 4-point response scales. While there was a slight preference for the 3-point scale based on stage one pilot testing, there was still interest in understanding if the 4-point scale might work with residents receiving memory care services. Interviewers used cue cards that numbered and displayed each answer option (e.g., 1 Always or Most of the time, 2 Some of the time, 3 Rarely or Never). Respondents could verbally reply with the number or words for their answer, or they could point or gesture to their answer. Stage two pilot testing used the same questionnaire as stage one. In addition to the 57 questionnaire items, respondents were asked open-ended items to assess overall comprehension and the questionnaire experience. Interviewers were asked to note any challenges, confusion, or memory issues that came up.

### Phase 2: Statewide pilot testing

Phase two of statewide pilot testing took place in two stages because of challenges with in-person data collection given the ongoing COVID-19 pandemic.

### Statewide pilot testing procedures

#### Stage 1

In September of 2021, statewide data collection began with the goal of testing the stability of the reliability and validity results found during phase one of pilot testing. Data collection was initially planned to rely on in-person interviews at up to 858 facilities that served eight or more residents, including those licensed with memory care. Initial targets were based on obtaining statistically representative samples at the facility level. Interviewers completed comprehensive training including classroom instruction and practical experience conducting supervised interviews. After training, experienced Quality Assurance Mentors observed interviewers, recorded resident responses independently for inter-rater reliability calculations, and provided feedback to interviewers.

There were many challenges with in-person data collection due to the ongoing COVID-19 pandemic. For example, facilities were experiencing outbreaks and would close to visitors. ALF workforce shortages were pronounced and there were not enough staff in some facilities to coordinate interviews. Many facilities opted out of participating, given these persistent issues. In-person interviews were completed at only 97 facilities before data collection goals were modified in January 2022.

#### Stage 2

During Stage 2 of statewide pilot testing (March – June 2022), the focus of the project shifted to maximize data collection while testing phone and mailed questionnaire administration. The goal for phone and mailed administrations was to obtain at least 400 questionnaires per mode to test for differences in questionnaire results by mode. With the shift in objectives, facilities with eight or more residents that had not participated in Stage 1 and did not have memory care licenses were eligible for Stage 2. Facilities with memory care licenses were excluded from Stage 2 given concerns over conducting interviews via phone and mail with memory care residents. The remaining facilities were randomly assigned to phone or mail administration.

#### Mail mode

For mail administration, a box with questionnaire packets was mailed to the facility to then distribute. An letter accompanied each questionnaire describing the intent and voluntary nature of participation. By returning a complete questionnaire, residents consented to study participation. No resident-level sampling was conducted for facilities assigned to mailed administration.

#### Phone mode

For phone administration, facilities were assigned to a data collection window and trained interviewers were provided with resident lists. Interviewers called participating facilities every morning to obtain guardian refusal lists prior to beginning calls. When interviewers called potential respondents, they followed a script describing the questionnaire and asking for voluntary consent to participate. No resident-level sampling was conducted for facilities assigned to phone administration.

### Phase 2 pilot participants

The facility-level participation rate was lowest for in-person data collection (22%) as compared with phone and mail modes (50% each; Table 1). For each mode, participating facilities were of different sizes, ranging from those that had capacity for fewer than eight residents to those that had capacity for more than 50 residents. Participating facilities across all modes were distributed throughout the state in rural, suburban and urban areas. While facility-level participation rates were higher for phone and mail questionnaire modes, at the resident-level, participation was highest for in-person data collection such that 93% of residents approached were interviewed (*n* = 1,692). For phone and mail administration resident-level participation rates were 41% and 20%, respectively (*n* = 517 for each). Demographic information for residents by data collection mode is presented in Table 1.

**Table 1:** Demographics of respondents by data collection mode during phase 2 statewide pilot testing

Demographics	Stage 1	Stage 2	
	In-person	Phone	Mail
Average Age	85	79	83
Age Max. – Min.	20–103	22–81	23–105
Male	453 (28%)	168 (35%)	148 (29%)
Female	1,144 (70%)	317 (65%)	353 (68%)
White	1,325 (81%)	397 (82%)	468 (91%)
Black	9 (1%)	27 (6%)	16 (3%)
Hispanic/Latino/a	4 (< 1%)	5 (1%)	3 (1%)
Middle Eastern/North African	2 (< 1%)	4 (1%)	0 (0%)
Native Hawaiian/Pacific Islander	4 (< 1%)	0 (0%)	0 (0%)
Asian	6 (< 1%)	0 (0%)	4 (1%)
American Indian/Alaskan Native	12 (1%)	8 (2%)	2 (< 1%)
Other Race/Ethnicity	193 (12%)	30 (6%)	0 (0%)
Lived in facility for < 1 year	586 (36%)	157 (32%)	173 (35%)
% Receiving memory care services	9%	Not applicable	Not applicable

### Data analysis

For each phase of data collection, we conducted detailed descriptive analyses and exploratory factor analyses. Analyses were conducted separately for each phase. Descriptive statistics were calculated to understand variability in responses and use of the full response scale as well as the extent to which residents could answer each of the items on the questionnaire by examining ‘Not Applicable/Don’t Know’ and missing responses. To analyze cognitive testing results with residents in memory care, descriptive statistics were calculated with a focus on comparing the 3- versus 4-point response scales. Additionally, open-ended responses were characterized thematically.

Exploratory factor analysis (principal axis factoring) with varimax rotation was conducted to understand the underlying factor structure of the items. This method was used because it assumes that total variance is comprised of common and unique variance among items. The goal was also to understand any latent constructs that defined the relationships among items. Missing data was handled by substituting the mean of answered items within a sub-domain when just one item in any given sub-domain was missing. Reliability was assessed by calculating Cronbach’s alpha for each of the identified factors. A Cronbach’s alpha of 0.70 or higher is considered acceptable [15]. In addition, a comparison of item-level correlations for items within sub-domains to that of items outside of the sub-domain revealed that the strength of correlations of items within sub-domains was higher than correlations with items in other sub-domains. To assess possible differences in results as a function of questionnaire administration mode we compared average ratings for each factor by mode using one-way analyses of variance (ANOVA) and the Scheffe post hoc test.

To further assess construct validity, we compared mean-level ratings of each factor across different ratings of the selected indicators by conducting ANOVA’s using a p-value of < 0.01 to determine statistical significance (see supplemental materials for the indicators). We hypothesized that more positive ratings of most indicators would be associated with higher factor scores. For example, we hypothesized that residents who graded the facility higher would also report higher satisfaction of the staff, dining, safety and privacy, etc. We also hypothesized that increased contact with family and loved ones would be associated with higher scores on multiple factors. We hypothesized that residents who did not use mobility devices or need help with activities of daily living would have higher levels of satisfaction across factors. Finally, for demographics such as gender, we hypothesized there would be no differences in satisfaction as a function of gender.

## Results

### Phase 1 targeted pilot testing

#### Mailed questionnaires

##### Descriptives

Item level descriptive results (*n* = 441) are briefly discussed here (supplemental materials have detailed results). Results tended to be positive; respondents answered ‘Yes, always/most of the time’ or ‘Yes, some of the time’ most often. The full response scale was used across all items with respondents also indicating ‘No, rarely or never’ or ‘Not applicable/don’t know’ although at lower rates. Rates of missingness ranged from < 1%-6%. Overall ratings were used to assess general perceptions of QOL and facility quality. Here too, responses were positive, yet there were responses across each of the full scales.

##### Factor analysis and reliability

Results (see supplemental materials) were based on 254 complete responses. The initial solution was a 10-factor solution. However, in examining the scree plot 7-, 8-, and 9-factor solutions were explored with the 7-factor solution resulting in the best fit. Each of the seven factors had an eigen value > 1.0. The seven-factor solution explained 54.56% of the total variance and the scree plot of the seven-factor solution indicated a leveling off after the seventh factor. The first factor explained 26.12% of the variance while the second and third factors explained 7.55% and 5.75%, respectively. The fourth and fifth factors explained 4.39% and 3.96% of the common variance, respectively. The seven resident QOL sub-domains identified in the factor analysis were found to have adequate internal consistencies evidenced by Cronbach’s alphas ranging from 0.63 to 0.86.

#### Memory care cognitive testing

Across each domain, responses were more varied with the 3- versus 4-point response scale. Responses on the 4-point scale tended to skew slightly more positively. Additionally, ‘Not Applicable’ and ‘Nonresponse’ answers were more prevalent when the 4-point scale was used. Many residents responded to questions with ‘yes’ or ‘no’ initially, leading to items and/or response scales being repeated frequently. Therefore, the response scale was adjusted to a 3-point scale and the words ‘how often’ were added to each item for phase two of pilot testing.

Residents receiving memory care services generally felt positively about the interview and its length. For a few demographic items, (e.g., how long the resident had lived at the facility) challenges with memory were evident to the interviewers, so they were modified.

### Phase 2 statewide pilot testing

#### Differences by mode

We compared average ratings for each factor by administration mode to see if there were systematic and statistically significant differences in ratings as a function of mode (results available upon request). No pattern of statistically significant differences was evident. There were dramatically different response rates as a function of mode: 93% for in-person interviewing, 41% for phone and 20% for mailed administration.

#### Factor analysis

Factor analysis results for phase two are presented in Table 2. All questionnaires across modes and facilities of all sizes were included. Missing data was handled such that if one item was missing in any domain that had more than three items, the missing data was imputed based on the average of the other items answered in that domain. Ten items were omitted from the factor analysis due to a high percentage of not applicable and missing data: six items regarding activities and two about finances were not included because there were anchor questions prior to these items asking if respondents were involved in activities and their own finances. If the respondent answered no, they were to skip those items resulting in a significant amount of not applicable data. Two additional items were omitted because more than 20% of responses were missing, don’t know, not applicable, or no response.

**Table 2:** Rotated factor matrix results for resident quality of life questionnaire (*N* = 1,776)

Minnesota Assisted Living Quality of Life Questionnaire	Factor loading				
	1	2	3	4	5
**Factor 1: The People Who Work Here**					
How often do the people who work here follow through when you have a complaint or a problem?	**0.62**	0.24	0.16	0.13	0.06
How often are you confident the people who work here can address your healthcare needs?	**0.60**	0.17	0.23	0.22	0.04
How often do the people who work here come quickly when you need help?	**0.58**	0.19	0.19	0.18	0.02
How often do you get enough help with your everyday activities if you need it?	**0.53**	0.19	0.14	0.12	0.03
How often are you confident the people who work here know what to do if you have a medical emergency?	**0.53**	0.19	0.19	0.31	0.04
How often do you feel comfortable asking for help when you need it?	**0.49**	0.14	0.34	0.12	0.08
How often do the people who work here treat you with respect?	**0.47**	0.13	0.46	0.02	0.21
How often do the people who work here try to get to know you?	**0.45**	0.15	0.12	0.20	0.10
How often are the services you receive here provided the way you want?	**0.37**	0.27	0.23	0.35	0.11
**Factor 2: Food**					
How often do you like the food served here?	0.18	**0.72**	0.22	0.06	0.01
How often is there enough variety in the meals offered here?	0.17	**0.69**	0.10	0.13	0.10
How often do you have enough choice in the meals offered here?	0.17	**0.66**	0.08	0.17	0.12
How often do you look forward to mealtimes here?	0.20	**0.64**	0.21	0.11	0.03
How often can you eat your meals when you want to?	0.16	**0.43**	0.03	0.11	0.06
How often does [insert facility name] offer access to healthy foods, like fruits and vegetables, if you want them?	0.16	**0.43**	0.19	0.14	0.01
**Factor 3: Security**,** Safety & Privacy**					
How often do you feel you have enough privacy here?	0.14	0.13	**0.55**	0.26	0.02
How often do you feel safe here?	0.14	0.12	**0.53**	0.13	0.09
How often are your personal belongings safe here?	0.21	0.14	**0.47**	0.20	0.03
How often is it quiet enough for you to sleep here?	0.13	0.09	**0.39**	0.12	0.07
How often do the people who work here ask to come in before entering your room?	0.06	0.10	**0.36**	0.29	0.03
How often do you feel comfortable voicing a complaint or concern?	0.24	0.12	**0.29**	0.24	0.06
How often do the people who work here ever get angry at you?	0.16	0.08	**0.29**	0.03	0.08
How often are the common areas well maintained?	0.25	0.14	**0.28**	0.16	0.11
**Factor 4: Choice/Autonomy**					
How often are you allowed to personalize your room?	0.12	0.05	0.22	**0.45**	0.06
How often can you decide how to spend your time each day?	0.09	0.13	0.18	**0.40**	0.11
How often are you as involved in decisions about the services you receive here as you want to be?	0.26	0.22	0.09	**0.39**	0.13
How often do you spend as much time outdoors as you would like?	0.16	0.11	0.04	**0.39**	0.12
How often are there places for residents to socialize with other residents?	0.23	0.11	0.20	**0.30**	0.17
Do you have friends here?	0.12	0.08	0.09	**0.26**	0.12
**Factor 5: Religion/Spirituality**					
How often are the people who work here respectful of your religious or spiritual practices?	0.10	0.08	0.20	0.22	**0.66**
How often are there enough opportunities for you to practice your religious or spiritual beliefs here?	0.08	0.12	0.08	0.28	**0.59**

Note: All items used a 3-point response scale ranging from 1 (No, rarely or never) to (Yes, always/most of the time)

The initial solution using principal axis factoring with a varimax rotation (*n* = 1,776) resulted in seven factors. However, in examining the scree plot, eigenvalues, and variance explained, six- and five-factor solutions were also explored with five factors resulting in the best fit, the most meaningful solution, and explaining 44.61% of the variance. The first factor explained 25.77% of the variance while the second and third factors explained 6.03% and 4.96%, respectively. The fourth and fifth factors explained 4.33% and 3.52% of the common variance, respectively. A factor loading rounded to 0.30 was considered moderately correlated and thus all items were retained [16]. While items related to meaningful activities and finances were excluded from the factor analysis, they were included in additional analyses.

Correlations among extracted factors were calculated (Table 3). Most correlations were positive, yet negligible to low. Correlations between ‘The People Who Work Here’ and ‘Safety, Security, and Privacy’ and between ‘Choice/Autonomy’ and ‘Religion/Spirituality’ were positive and moderate.

**Table 3:** Correlations among extracted factors after varimax rotation

Factor	*n*	M	SD	1	2	3	4	5
1. The People Who Work Here	2659	86.43	17.22	-				
2. Food	2540	75.59	24.46	0.48*	-			
3. Security, Safety, & Privacy	2728	92.28	11.92	0.53*	0.38*	-		
4. Choice/ Autonomy	2671	84.61	16.12	0.49*	0.37*	0.46*	-	
5. Religion/ Spirituality	2600	88.49	23.44	0.27*	0.25*	0.29*	0.53*	-

* *p* < .001

##### Reliability

The resident QOL questionnaire factors had adequate to high internal consistencies evidenced by Cronbach’s alphas ranging from 0.60 to 0.85 for each of the five factors in addition to items conceptually related to activities and finances (Table 4).

**Table 4:** Cronbach’s alphas for each factor

Factor	Cronbach’s alpha	Number of Items
The People Who Work Here	0.85	9
Food	0.82	6
Security, Safety, & Privacy	0.69	8
Choice/Autonomy	0.60	6
Religion/Spirituality	0.71	2
Activities	0.77	6
Finances	0.55	2

Item-level correlations for items within sub-domains were compared to item-level correlations with items outside of the sub-domain (i.e., item-total correlations; results available if requested), but the strength of correlations of items within sub-domains was higher than correlations with items in other sub-domains indicating that items within domains are more related to each other than to items in other domains.

We also compared average ratings of factors across different levels of global ratings such as the facility grade, residents’ self-reported QOL, and for a few demographic variables like gender. We expected that more positive ratings of global quality indicators would be associated with higher factor scores, which was supported by the analyses. Residents who rated their facility as “A- Excellent” had mean level scores on each factor that were significantly higher than the factor ratings for residents who rated their facility as a C or lower. Higher self-reported ratings of QOL, health, and memory were related to higher satisfaction across factors. Additionally, residents who reported speaking with or seeing family members or friends less often and those who needed help with activities of daily living were less satisfied across sub-domains. There were no differences by gender and minimal differences when considering the length of time residents had lived at facilities. Finally, residents with memory care services tended to have lower levels of satisfaction across factors as compared with residents not receiving memory care services.

## Discussion

This study had two objectives: (1) systematically develop and test a questionnaire assessing the QOL for residents of ALFs, and (2) assess the psychometric properties of the questionnaire. Results from statewide pilot testing showed the questionnaire had good internal consistency, validity, and reliability. Sub-scales of the questionnaire demonstrated convergent validity by positively correlating with related indicators of quality (e.g., overall rating of facility), supporting construct validity of the questionnaire [4]. Thus, the study presents not only a viable, holistic measure to assess QOL among assisted living residents on a large scale but also presents a roadmap for researchers and practitioners looking to develop QOL measures for residents of long-term care facilities. The creation of this QOL questionnaire also fills an important gap in quality measurement in assisted living settings since other existing tools are limited in scope and application. Directly assessing QOL through self-reported surveys is the optimal way to assess the internal states of individuals [2, 6, 12]. With few existing instruments that assess QOL across multiple sub-domains among assisted living residents [7], the study contributes to the literature by presenting a QOL questionnaire that was robustly developed and psychometrically tested with a large sample.

Findings demonstrate the feasibility of administering this questionnaire to ALF residents and suggest that other U.S. states could administer this or similar questionnaires to standardize QOL assessments and track ALF quality. Further, because the questionnaire includes several sub-scales that measure different sub-domains of quality, it could link sub-domains of quality with other factors, such as active aging determinants and individual or facility characteristics, which can then inform quality improvement efforts within ALFs [8, 12]. While there were no systematic differences in responses by administration mode, pilot testing made clear that face-to-face administration was the most inclusive mode that yielded the highest response rate. Including residents with varying diagnoses, levels of independence, and those who receive memory care services is essential to ensure representative data. Researchers and practitioners should elect to use the administration mode(s) that maximizes the response rate, while maintaining in-person administration for ALF residents receiving memory care services and those with varying levels of cognitive impairment or communication challenges.

The resident QOL questionnaire continues to be administered annually, statewide in Minnesota. Additional years of data collection have resulted in minor adjustments to the questionnaire. For example, some demographic items have been revised to reduce the survey length. Additionally, Minnesota has incorporated this questionnaire as one metric in their comprehensive Assisted Living Report Card (https://alreportcard.dhs.mn.gov/), which aims to help consumers select the best long-term care facility based on several quality measures. Resident QOL is one aspect consumers can view across facilities along with resident safety, family satisfaction, staffing, licensing, and resident health.

Although we developed and tested a new questionnaire to assess QOL among assisted living residents, there are some limitations to note. The questionnaire was tested within Minnesota but did not include residents in tribal lands and therefore should be tested in other U.S. states, tribal lands, and internationally to determine applicability. The questionnaire development and testing process was impacted by the onset of the COVID-19 pandemic, which required the research team to pivot data collection strategies. Since the questionnaire was completed by participants in English, future testing should include translated versions of the questionnaire. Finally, the use of a 3-point scale may limit variability in responses and ultimately in facility-level scores. However, using a 3- versus 4-point scale allowed us to include a broader group of assisted living residents, particularly residents who receive memory care services. Overall, future research on this questionnaire could focus on replicating the results we present using additional years of resident data to document the stability of the results presented here. Furthermore, additional research to understand the extent to which and how facility administrators and staff use these results to inform quality improvement efforts and how consumers use these results to make decisions about long-term care options could inform how results are presented and to maximize data use.

## Conclusion

Reliable and validated instruments to measure QOL among assisted living residents continue to be limited in scope. This study presents not only a viable, holistic measure to assess QOL among assisted living residents on a large scale, but also a roadmap for researchers and practitioners looking to develop QOL measures for residents of long-term care facilities. This questionnaire presents an opportunity for other localities and states to consider implementing a similar measure to help inform consumers as they and their family members evaluate long-term care options and provide information to facilities to improve QOL for their residents.

## Supplementary Information

Below is the link to the electronic supplementary material.

Supplementary Material 1

## Data Availability

The datasets generated and analyzed during the current study are not publicly available due funder restrictions but are available from the corresponding author on reasonable request.

## Abbreviations

ALF: Assisted living facility
ANOVA: Analysis of Variance
DHS: Department of Human Services
QOL: Quality of life
