# Supplementary material for: Assisted living resident quality of life questionnaire: development and validation
Source: J Patient Rep Outcomes. 2026 Jan 24;10:26. doi: 10.1186/s41687-026-00994-6 (PMC12913808; doi:10.1186/s41687-026-00994-6)
Supplement: Supplementary file 1 — Supplementary Material 1 [file 41687_2026_994_MOESM1_ESM.docx]

**Supplementary Materials**

**Table S1**

*Factor Analysis Results for Phase One Target Pilot Testing*

| Questionnaire Item | Factor | | | | | | |
| --- | --- | --- | --- | --- | --- | --- | --- |
|  | 1 | 2 | 3 | 4 | 5 | 6 | 7 |
| Are you confident the people who work here can address your healthcare needs? | **0.70** | 0.22 | 0.09 | 0.07 | 0.15 | 0.15 | 0.14 |
| Do the people who work here follow through when you have a complaint or problem? | **0.65** | 0.11 | 0.17 | 0.09 | 0.29 | 0.15 | 0.13 |
| Do you get enough help with your everyday activities if you need it? | **0.61** | 0.12 | 0.11 | 0.07 | 0.12 | 0.18 | 0.07 |
| Are you confident the people who work here know what to do if you have a medical emergency? | **0.60** | 0.15 | 0.09 | 0.08 | 0.10 | 0.22 | 0.11 |
| Do you feel comfortable asking for help when you need it? | **0.56** | 0.02 | 0.01 | 0.10 | 0.07 | 0.09 | 0.12 |
| Do the people who work here come quickly when you need help? | **0.55** | 0.05 | 0.23 | 0.02 | 0.20 | 0.04 | 0.08 |
| Are there places for residents to socialize with other residents? | **0.38** | 0.25 | 0.05 | 0.17 | -0.01 | 0.02 | 0.11 |
| Do the people who work here ever get angry at you? | **0.21** | 0.02 | 0.01 | 0.01 | -0.01 | 0.08 | -0.01 |
| Do you have enough choice in the meals offered by the facility? | 0.07 | **0.82** | 0.14 | 0.10 | 0.05 | 0.16 | 0.09 |
| Is there enough variety in the meals offered here? | 0.12 | **0.82** | 0.22 | 0.08 | 0.06 | 0.07 | -0.00 |
| Do you like the food served here? | 0.10 | **0.79** | 0.21 | -0.01 | 0.01 | 0.03 | 0.10 |
| Do you look forward to mealtimes? | 0.18 | **0.66** | 0.12 | 0.03 | 0.12 | 0.02 | 0.09 |
| Does the facility offer access to healthy foods, like fruits and vegetables, if you want them? | 0.05 | **0.45** | 0.22 | 0.18 | 0.15 | 0.25 | 0.08 |
| Can you eat your meals when you want to? | 0.20 | **0.40** | 0.08 | 0.06 | -0.01 | 0.15 | 0.13 |
| Is there enough variety in the activities here? | 0.12 | 0.18 | **0.77** | 0.23 | 0.08 | 0.12 | -0.03 |
| Do you enjoy the way you spend your time most days? | 0.17 | 0.21 | **0.62** | 0.04 | 0.15 | 0.20 | 0.38 |
| Do you have enough activities to keep your mind active? | 0.06 | 0.19 | **0.58** | 0.12 | -0.05 | 0.04 | 0.17 |
| Are there things to do here on the weekends that you enjoy? | 0.18 | 0.33 | **0.55** | 0.08 | 0.13 | 0.06 | 0.09 |
| Do you like the activities that are provided here? | 0.15 | 0.21 | **0.52** | 0.17 | 0.12 | 0.13 | 0.24 |
| Are the people who work here respectful of your religious or spiritual practices? | 0.16 | 0.14 | 0.18 | **0.78** | 0.07 | 0.09 | 0.10 |
| Are the people who work here respectful of your culture? | 0.05 | -0.00 | 0.10 | **0.72** | 0.31 | 0.14 | 0.12 |
| Are there enough opportunities for you to practice your religious or spiritual beliefs here? | 0.14 | 0.14 | 0.24 | **0.62** | 0.04 | 0.11 | 0.19 |
| Is it quiet enough for you to sleep here? | 0.16 | -0.01 | 0.10 | 0.112 | **0.71** | 0.04 | 0.04 |
| Are the common areas well maintained? | 0.23 | 0.13 | 0.08 | 0.07 | **0.64** | 0.18 | -0.01 |
| Do the people who work here treat you with respect? | 0.26 | 0.16 | 0.03 | 0.19 | **0.63** | 0.20 | 0.22 |
| Are you satisfied with how your mediations are managed? | 0.39 | 0.08 | 0.14 | -0.05 | 0.19 | **0.56** | -0.13 |
| Do you feel safe here? | 0.15 | 0.09 | 0.06 | 0.30 | 0.34 | **0.55** | 0.07 |
| Do you feel comfortable voicing a complaint or concern? | 0.27 | 0.20 | 0.18 | 0.04 | 0.04 | **0.45** | 0.23 |
| Do you feel you have enough privacy? | 0.19 | 0.09 | -0.03 | 0.21 | 0.15 | **0.42** | 0.28 |
| Are your personal belongings safe here? | 0.35 | 0.16 | 0.10 | 0.04 | 0.26 | **0.41** | 0.10 |
| Do the people who work here ask to come in before entering your room? | 0.23 | 0.09 | 0.08 | 0.08 | 0.02 | **0.34** | 0.04 |
| Are you as involved in decisions about the services you receive here as you want to be? | 0.19 | 0.23 | 0.22 | 0.27 | 0.03 | **0.30** | 0.26 |
| **Are you allowed to personalize your room?** | 0.08 | 0.05 | 0.14 | 0.22 | -0.19 | **0.29** | **0.24** |
| Can you decide how to spend your time each day? | 0.02 | 0.09 | 0.16 | 0.18 | 0.02 | 0.17 | **0.57** |
| **Do the people who work here try to get to know you?** | **0.36** | 0.14 | -0.07 | 0.01 | 0.23 | 0.02 | **0.39** |
| Do you spend as much time outdoors as you would like? | 0.01 | 0.23 | 0.26 | 0.05 | -0.11 | 0.03 | **0.37** |
| Are the services you receive here provided the way you want? | 0.30 | 0.11 | 0.11 | 0.24 | 0.12 | 0.29 | **0.35** |
| Do you have friends here? | 0.14 | -0.01 | 0.12 | 0.05 | 0.11 | -0.02 | **0.31** |
| Do you feel included in things that are happening here? | 0.19 | 0.19 | 0.25 | 0.20 | 0.03 | 0.13 | **0.31** |

*NOTE: Items that load onto more than one factor are bolded.

**Additional Analysis to Assess Construct Validity**

Additional analyses were performed to further assess the construct validity of the resident quality of life questionnaire. Scores for each of the five factors plus the items related to activities and finances were assessed against the following indicators:

1. The grade residents gave the facility on a 5-point scale from A-Excellent to F-Failing
2. Resident report of their own quality of life on a 5-point scale from Excellent to Poor
3. Resident rating of their own health status on a 5-point scale from Excellent to Poor
4. Resident rating of their memory on a 5-point scale from Excellent to Poor
5. How often residents see or talk with family or friends who do not live at the facility ranging from Everyday to Less than Monthly
6. Residents receiving help with activities of daily living (Yes/No)
7. Resident gender (Male/Female/Not Listed)
8. Length of time living at the facility (Less than or More than One Year)
9. Whether the resident lived in memory care (based on interviewer observation for in-person data collection only)
